# Rapid Diagnosis of Staphylococcal Catheter-Related Bacteraemia in Direct Blood Samples by Real-Time PCR

**DOI:** 10.1371/journal.pone.0161684

**Published:** 2016-08-29

**Authors:** Yuliya Zboromyrska, Cristina De la Calle, Marcelo Soto, Laura Sampietro-Colom, Alex Soriano, Míriam José Alvarez-Martínez, Manel Almela, Francesc Marco, Ruth Arjona, Nazaret Cobos-Trigueros, Laura Morata, José Mensa, José Antonio Martínez, Aurea Mira, Jordi Vila

**Affiliations:** 1 Department of Clinical Microbiology, Biomedical Diagnostic Centre (CDB), Hospital Clínic, School of Medicine, University of Barcelona, Barcelona, Spain; 2 Department of Infectious Diseases, Hospital Clínic, University of Barcelona, Barcelona, Spain; 3 Health Technology Assessment Unit, Hospital Clínic, University of Barcelona, Barcelona, Spain; 4 ISGlobal, Barcelona Centre for International Health Research (CRESIB), Hospital Clínic, University of Barcelona, Barcelona, Spain; 5 CDB, Hospital Clínic, School of Medicine, University of Barcelona, Barcelona, Spain; University Medical Center Groningen, NETHERLANDS

## Abstract

Catheter-related bacteremia (CRB) is an important cause of morbidity and mortality among hospitalized patients, being staphylococci the main etiologic agents. The objective of this study was to assess the use of a PCR-based assay for detection of staphylococci directly from blood obtained through the catheter to diagnose CRB caused by these microorganisms and to perform a cost-effectiveness analysis. A total of 92 patients with suspected CRB were included in the study. Samples were obtained through the catheter. Paired blood cultures were processed by standard culture methods and 4 ml blood samples were processed by GeneXpert-MRSA assay for the detection of methicillin-susceptible (MSSA) or methicillin-resistant (MRSA) *Staphylococcus aureus*, and methicillin-resistant coagulase-negative staphylococci (MR-CoNS). Sixteen CRB caused by staphylococci were diagnosed among 92 suspected patients. GeneXpert detected 14 out of 16 cases (87.5%), including 4 MSSA and 10 MR-CoNS in approximately 1 hour after specimen receipt. The sensitivity and specificity of GeneXpert were 87.5% (CI 95%: 60.4–97.8) and 92.1% (CI 95%: 83–96.7), respectively, compared with standard culture methods. The sensitivity of GeneXpert for *S*. *aureus* was 100%. Regarding a cost-effectiveness analysis, the incremental cost of using GeneXpert was of 31.1€ per patient while the incremental cost-effectiveness ratio of GeneXpert compared with blood culture alones was about 180€ per life year gained. In conclusion, GeneXpert can be used directly with blood samples obtained through infected catheters to detect *S*. *aureus* and MR-CoNS in approximately 1h after sampling. In addition, it is cost-effective especially in areas with high prevalence of staphylococcal CRB.

## Introduction

Catheter-related bloodstream infection (CRBSI) is an important cause of morbidity and mortality among hospitalized patients. Staphylococci are the main aetiological agents of CRBSI [1]. Bacteremia due to *Staphylococcus aureus* is a severe disease with a high risk of complications and a high mortality rate [2]. In addition, coagulase-negative staphylococci (CoNS) are also important aetiological agents of CRBSI [3]. Early adequate antibiotic treatment of staphylococcal bacteremia is associated with a better prognosis [4] and early removal of the catheter reduces the risk of developing haematogenous complications [5]. However, the conventional blood culture (BC) method usually requires more than 16 h for bacterial growth detection in the case of staphylococci [6]. To address this problem, several molecular assays have been developed in recent years for direct detection of pathogens in whole blood samples [7]. Although these assays provide results more rapidly, the low bacterial density in blood during bacteremia (1–10 CFU/mL) often results in low sensitivity [8, 9]. However, the intraluminal density of bacteria in CRBSI cases is high (>1,000 CFU/ml) [10–12], which explains the shorter time-to-positivity (TTP) of BC obtained from a catheter compared to those from a peripheral vein [13]. We therefore hypothesized that a rapid Real-Time Polymerase Chain Reaction (rt-PCR)-based assay performed in blood obtained through the infected catheter would have a high sensitivity for detecting the presence of microorganisms causing CRBSI. The main objective of this study was to assess the use of the GeneXpert MRSA/SA BC assay (Cepheid, Sunnyvale, USA) to detect methicillin-susceptible (MSSA), methicillin-resistant (MRSA) *S*. *aureus*, and methicillin-resistant CoNS (MR-CoNS) directly from blood obtained through the catheter and to perform a cost-effectiveness analysis comparing this new test to the traditional BC method.

## Materials and Methods

### Sample Collection

From October 2012 to October 2014 we collected 92 whole blood samples from 92 patients in whom CRBSI was suspected during hospitalization in our centre, a 700-bed university hospital. All patients were prospectively followed until a definitive source of bacteremia was established. Criteria for suspecting CRBSI included: sudden onset of fever with local inflammatory signs at the catheter insertion site, fever without local signs or any other evident source of infection. In all cases, 4 ml of whole blood were obtained through the catheter and submitted to the Microbiology laboratory in a sterile EDTA-containing tube for the GeneXpert assay and blood samples for paired BC (about 10 ml for a single BC vial) were drawn sequentially from the catheter and a peripheral vein without waiting between draws. Patients were included in the study during morning working time, and GeneXpert was performed within 1 hour before sample collection for BC in all cases.

The criteria of the definitive diagnosis of CRBSI were: isolation of the same microorganism from BCs obtained from the catheter and peripheral vein with a differential TTP of ≥2 hours or the isolation of the same microorganism from both a catheter tip and at least one percutaneous BC [1].

Microorganisms isolated from positive BC were considered as contaminants if commensal bacteria were detected in only one set of paired BC or different CoNS were isolated from each set of BC and another source of fever was identified.

The study was approved by the Hospital Clinic of Barcelona Ethics Committee (study no. 2012/7798). Patient records were anonymized and de-identified prior analysis. No informed consent was considered necessary for this study.

### Routine microbiological techniques

BCs were collected and transported to the laboratory in less than 2 hours. BCs vials were incubated in BACTEC FX (Becton Dickinson, MD, USA) for a maximum of 120 h. In case of positivity, Gram staining and subculture on appropriate solid media were performed. The TTP of each BC bottle was recorded. If the catheter was removed, the Maki roll-on semiquantitative method for catheter tip culture was used. The final identification of bacterial species was achieved using matrix-assisted laser desorption/ionization time-of-flight mass spectrometry (MALDI-TOF MS) (Bruker Daltonics, Bremen, Germany). Routine antimicrobial susceptibility included the Phoenix system (Becton Dickinson, MD, USA) for *S*. *aureus* and the disk diffusion method for CoNS. Results of susceptibility testing were interpreted according to EUCAST guidelines (http://www.eucast.org).

### The GeneXpert assay

To recover bacteria from whole blood, the EDTA collection tube was centrifuged at 430 × g for 5 min and then the supernatant was centrifuged at 15,600 × g for 2 min. The pellet obtained was resuspended in 100 μl of sterile saline solution (0.9% NaCl) and used for the GeneXpert test according to the manufacturer's instructions. The GeneXpert used was Xpert MRSA-SA BC G3 Version 24. GeneXpert was considered positive for MR-CoNS if only the methicillin resistance gene (*mecA*) was detected; positive for MSSA if only the staphylococcal protein A (*spa*) target was detected; and positive for MRSA if the *spa*, *mecA* genes and the staphylococcal chromosome genomic island (SCC*mec*) were identified.

The current cycle threshold (Ct) cut-off values of the GeneXpert assay were established for positive BCs containing high bacterial concentrations, with a valid maximum cycle for all three targets of 36. Since the expected bacterial inoculum in whole blood is much lower, two bacterial strains of *S*. *aureus* (MSSA and MRSA) were used to study the analytical detection limits of GeneXpert assay. Four millilitres of whole blood were spiked with bacteria to achieve three different concentrations (10, 100 and 1000 CFU/ml). The GeneXpert assay was performed in duplicate for each *S*. *aureus* strain and each bacterial concentration. Taking into account the results of the detection limits study, prolonged Ct values (>36) were also considered as positive.

### Cost-effectiveness analysis

The information was not provided to the physician in charge of the patient. Therefore, we performed an analysis according to the sensitivity and specificity of the test and using previous literature about complications related with *S*. *aureus* catheter-related bacteremia [5].

A cost-effectiveness analysis (CEA) of using GeneXpert to detect CRBSI caused by *S*. *aureus* and MR-CoNS was performed. The comparator was the conventional BC method. The main clinical outcome considered was the expected number of life years gained. Costs were measured from the perspective of our hospital and were obtained from hospital sources. Differences in costs and life years between testing strategies were used to compute the incremental cost-effectiveness ratio (ICER).

**Fig 1:**
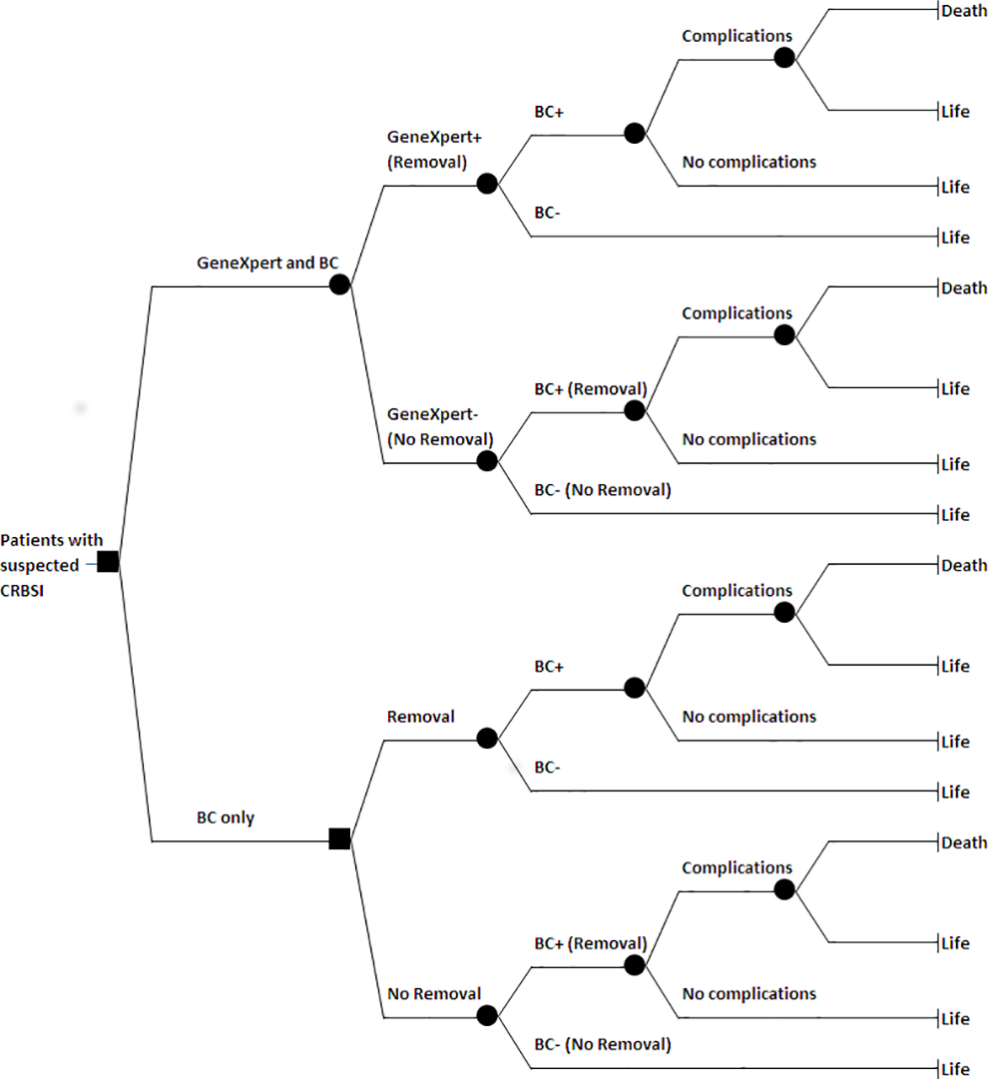
Decision tree for cost-effectiveness analysis. CRBSI, catheter-related bloodstream infection; BC, blood culture; -, negative; +, positive.

A decision tree based on the standard clinical approach to CRBSI was developed. The tree was used to model outcomes following different strategies (Fig 1). Individuals enter the model as patients with suspected CRBSI. All patients are tested with conventional BC. Under the standard protocol (lower branch of the tree) all patients receive empirical wide-spectrum antibiotics and a fraction of patients have the catheter removed before BC results are available. The removal decision is initially based solely on clinical criteria. In patients tested with GeneXpert (upper branch) the catheter is removed depending on the test result, which is known before BC results. The model assumes that a positive test leads to immediate catheter removal and patients are treated with specific antibiotics.

Variables determining the probability of each health outcome are: the prevalence rate of *S*. *aureus* and MR-CoNS among suspected patients, the sensitivity and specificity of the GeneXpert test, the probability of haematogenous complications when the catheter is removed on suspicion of CRBSI and when catheter removal is delayed until BC results are available, and the probability of death related to CRBSI. The S1 File provides a detailed description of the economic model and data sources.

Resources considered for the cost analysis included BC costs, the GeneXpert kit, technical staff, catheters, wide-spectrum and specific antibiotics; imaging (abdominal ultrasound, transthoracic echocardiogram, and PET scan) and the use of an intensive care unit in case of haematogenous complications. Treatment of co-morbidities or CRBSI caused by bacteria other than staphylococci was not considered in the cost analysis. One-way deterministic and multivariate probabilistic sensitivity analyses were performed to evaluate the robustness of CEA.

### Statistical analysis

Statistical analysis was performed using SPSS software (SPSS, Chicago, IL). Differences were considered significant with a *P*< 0.05.

## Results

The results of the analytical detection limits study were as follows: samples with 10 CFU/ml bacterial concentration were negative by GeneXpert for the three targets (*spa*, *mecA*, SCC*mec*); samples with 100 CFU/ml were positive but with prolonged Ct values (>36); samples with 1000 CFU/ml were positive with recommended Ct cut-off values (≤36) for the three targets.

We evaluated 92 blood samples obtained from 92 patients and processed with paired BCs and GeneXpert. The source of bacteremia was presumed to be a short peripheral intravenous catheter in 3 patients (3.2%), a peripherally inserted central catheter in 18 (19.6%), a central subclavian or jugular venous catheter in 63 (68.5%), and a tunnelled catheter in 8 patients (8.7%). The median (IQR) length of stay of patients was 13 (5–22) days and the median catheter dwell time was 12 (7–23) days.

BCs were positive in 34 patients. Eighteen cases did not fulfil the CRBSI definition. In 15 cases, the microorganisms isolated (14 CoNS and 1 viridans group *Streptococcus*) were considered contaminants. None of the 14 contaminant CoNS was detected by GeneXpert. No specific treatment was prescribed in these cases. Two patients had bacteremia by Gram-negative bacilli, with the suspected source being a urinary tract infection in both; and the other patient had bacteremia due to *Enterococcus faecium* from an unknown source.

A total of 16 CRBSI were diagnosed among 92 suspected patients, therefore, the prevalence of staphylococcal CRBSI in our study population was 17.4%. Among these 16 CRBSI no septic metastasis and no death related was observed. GeneXpert detected 14 out of 16 cases (87.5%), including 4 MSSA and 10 MR-CoNS. Table 1 shows the concordance between definitive CRBSI cases and GeneXpert results. There were 2 false negative GeneXpert results, both caused by MR-CoNS.

**Table 1:** Comparison of results obtained by GeneXpert and convencional BC.

	No. of patients		
	Positive BC (%)	Negative BC (%)	Total (%)
**GeneXpert positive**	14 (15.2)^a^	6 (6.5)^b^	20 (21.7)
**GeneXpert negative**	2 (2.2)	70 (76.1)^c^	72 (78.3)
**Total (%)**	16 (17.4)	76 (82.6)	92 (100)

BC, blood culture

^a^ Including 4 *Staphylococcus aureus* detected.

^b^ Including 3 *S*. *aureus* detected.

^c^ Including 14 contaminant CoNS.

The median TTP in the BC of the 10 MR-CoNS detected by GeneXpert was 16.6h (IQR: 12.1–17.8), whereas the TTP of two MR-CoNS not detected by GeneXpert was 20.8 h and 24.1 h, respectively. Interestingly, the median TTP of not detected CoNS, including 14 contaminants and 2 false negatives, was 25.5 h (IQR: 20.0–41.8). The difference between the median TTP of MR-CoNS detected by GeneXpert (16.6 h) and those not detected (25.5 h) was statistically significant (*P* = 0.005).

On the other hand, GeneXpert identified 6 additional staphylococci (presumably false positives), including 3 MR-CoNS, 2 MSSA, and one sample, which offered two possibilities since the genes *mecA* and *spa* but not SCC*mec* were detected. These two possibilities are: 1) that both MR-CoNS and MSSA were present or 2) a MRSA strain with a SCC*mec* variant that was not detected. Indeed, the last option seems more plausible since the Ct values for both *mecA* and *spa* genes were similar with 37.7 and 37.5, respectively. Interestingly, 4 out of these 6 (66.7%) false positives were receiving active antibiotic treatment initiated at least 24 h before sampling. Briefly, one patient with MSSA was treated with ceftriaxone, the other patient with MSSA with meropenem and vancomycin; one patient with mixed MSSA and MR-CoNS was receiving meropenem and linezolid, and the other with MR-CoNS, received vancomycin. In contrast, among the 14 patients with GeneXpert results concordant to CRBSI diagnosis, only 2 (2/14, 14.3%) patients were receiving correct antimicrobial treatment for at least 24 h before sampling (*P* = 0.037).

The sensitivity, specificity, positive predictive value, and negative predictive value of GeneXpert in the present study were 87.5% (CI 95%: 60.4–97.8), 92.1% (CI 95%: 83–96.7), 70% (CI 95%: 45.7–87.2) and 97.2% (CI 95%: 89.4–99.5), respectively. The sensitivity, specificity, positive predictive value, and negative predictive value of GeneXpert for *S*. *aureus* were 100% (CI 95%: 39.6–100), 96.6% (CI 95%: 89.7–99.1), 57.1% (CI 95%: 20.2–88.2) and 100% (CI 95%: 94.6–100), respectively. The sensitivity of GeneXpert for MR-CoNS was 83.3%.

### Cost-effectiveness results

#### Clinical outcomes

Using prevalence figures in the hospital and mortality rates published in the literature[14], the probability of death for patients with suspected CRBSI was estimated at 2.59% for patients tested with conventional BC alone and 1.59% for patients tested with GeneXpert (Table 2). Taking life expectancy of the general population in Spain as reference, the average discounted life expectancy (with a 3% discount rate) for patients tested with BC and with GeneXpert were 17.01 and 17.18 years, respectively. The use of GeneXpert allowed earlier detection of 14 cases of CRBSI among 92 suspected patients (15.2%).

**Table 2:** Cost-effectiveness results (base case).

	GeneXpert and BC	Only BC	Difference
**Clinical outcomes**			
Death probability	1.59%	2.59%	-1.00%
Expected Life years (discounted)	17.183	17.010	0.173
Early detections	15.2%	-	15.2%
**Costs**			
Test price (€)	67+60	60	67
Cost per patient (€)	411.5	380.4	31.1
**Cost-effectiveness (ICER)**			
Incremental cost (€) per life year gained (discounted)			179.5
Incremental cost (€) per early detected case			204.2

BC, blood culture; ICER, incremental cost-effectiveness ratio.

#### Costs and cost-effectiveness

Costs per test were 60€ for one BC (on average, 15€ for one BC vial) and 67€ for the GeneXpert test. In the baseline analysis the cost per patient were 380.4€ and 411.5€, respectively. As mentioned in the methods section, these numbers only take into account testing and treatment of CRBSI caused by *S*. *aureus* and MR-CoNS. Because the use of GeneXpert is associated with a lower probability of haematogenous complications due to early detection and, therefore, lower treatment costs, the incremental cost of using GeneXpert is only of 31.1€ per patient. The incremental cost-effectiveness ratio (ICER) of GeneXpert compared with BC alone was about 180€ per life year gained. Similarly, the ICER per early detection was estimated at 204€.

**Fig 2:**
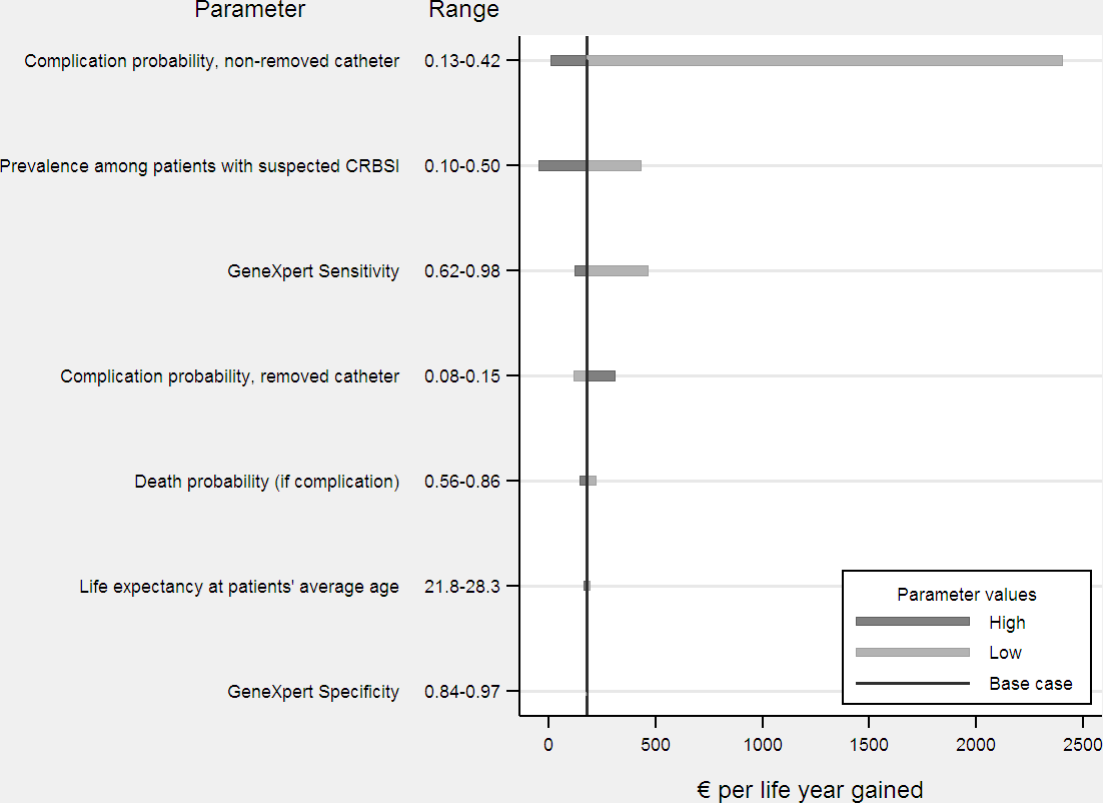
Univariate deterministic sensitivity analysis.

The sensitivity analysis showed that the probability of haematogenous complication when the catheter removal is delayed, the joint prevalence of *S*. *aureus* and MR-CoNS and the sensitivity of the GeneXpert test were the parameters that introduced the highest variability in the ICER (Fig 2). For complication probability in case of delayed catheter removal, the ICER for the GeneXpert test ranged between 9€ per life year gained (when the probability = 42%) and 2,405€ (when probability = 13%). As regards the influence of prevalence, the ICER for the GeneXpert test ranged between minus 46€ per life year gained (i.e., dominance of GeneXpert) when the prevalence rate was 50% and 435€ per life year gained when the prevalence was lower (i.e., 10%). Changes in sensitivity also affected ICER, which varied from 124€ to 470€ per life year gained for high (98%) and low (62%) sensitivity rates, respectively. The remaining parameters of the model (complication probability when the removal of the catheter is not delayed, the unconditional death probability for true positives, life expectancy, and specificity of GeneXpert) introduced less variability in the ICER.

This analysis addresses changes in incremental cost-effectiveness ratio (€/life year gained) when individual parameter values are varied. The vertical line corresponds to the base case incremental cost-effectiveness ratio of the model. CRBSI, catheter-related bloodstream infection

**Fig 3:**
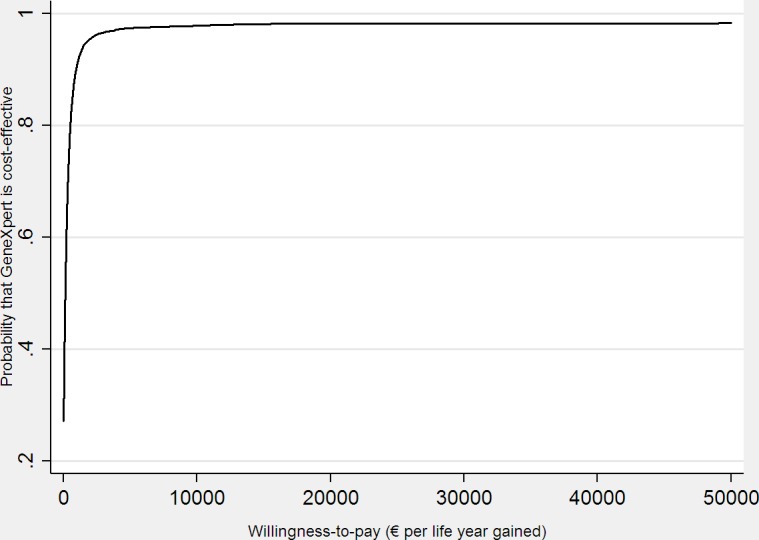
The probabilistic sensitivity analysis.

Results from the probabilistic sensitivity analysis are summarized in Fig 3. At a willingness-to-pay of 0€ per life year gained GeneXpert had a 27% probability of being cost-effective compared with BC. As the willingness-to pay increased, the probability that Genexpert was cost-effective quickly raised to 98%.

## Discussion

In this study, we demonstrated that GeneXpert is a useful and rapid test for the detection of *S*. *aureus* and MR-CoNS directly in blood samples obtained through an infected catheter. As GeneXpert results are available in approximately 1h, this test may help clinicians to early select the appropriate antibiotic treatment and to decide whether immediate catheter removal is necessary or not. A delay in the initiation of appropriate antibiotic therapy and prompt catheter removal are critical variables associated with the outcome of patients with CRBSI and the risk of developing septic metastases [5, 15]. In addition, as vancomycin treatment compared with a beta-lactam has been associated with a higher mortality rate in case of MSSA bacteremia, a rapid discrimination between MSSA and MRSA should improve the outcome of these patients.[16] Previously reported sensitivity of the GeneXpert from positive BC for MSSA and MRSA was 100% and 98.3%, respectively [17]. In the present study, the global sensitivity performed using blood obtained from an infected catheter was 87.5%, including MR-CoNS, and it increases up to 100% for MSSA.

According to a recent study from our institution, CoNS are the aetiology of 45% of CRBSI cases with a rate of methicillin resistance higher than 70% [18]. Interestingly, GeneXpert detects MR-CoNS in case of negative *spa* gene and positive *mecA*. To our knowledge, this is the first time that GeneXpert is used for this proposal and our results suggest that this is a promising diagnostic tool particularly because contaminants were not amplified. This could be attributed to the low bacterial density in contaminant cases as it was suggested by a significantly longer median TTP in concomitant BC of negative GeneXpert samples than of positive ones (25.5 h vs 16.6 h, *P* = 0.005). However, there were 2 true CRBSI due to MR-CoNS not detected by GeneXpert. In these cases the TTP was also ≥20 h and this is a limitation but it probably indicates less severe infections [19].

An interesting finding was the identification of six presumably false positive results. The most reasonable explanation is the previous exposure to active antibiotics, which inhibited their growth in culture media. However, we cannot rule out false positive results due to unspecific amplification.

Previously, the GeneXpert assay had been shown to have an important impact on the mean length of hospital stay and the mean hospital costs for bacteremic patients [20]. In another study, using GeneXpert results to guide anti-infective therapy proved to be less costly than empiric treatment for MRSA and also showed the potential to reduce mortality rates [21]. Regarding our results, the estimated ICER was only about 180€ per life year gained. Although there are no well-defined cost-effectiveness thresholds for life years gained, there are thresholds for quality adjusted life years (i.e., QALYs; range US $20,000/16,800€ and US $100,000/84,000€) [22]. The ICER estimated here is well below these figures; if any adjustment for quality were made the main results will not be affected.

There are several limitations in our study. First, the number of patients included was small and the study was performed in a single hospital. Second, as GeneXpert allows detection of only *S*. *aureus* and MR-CoNS, CRBSI caused by other etiological agents, including methicillin-susceptible CoNS, cannot be identified by this system. Third, results of the GeneXpert test were not taken into account for patients’ management; this fact and the low prevalence of *S*.*aureus* CRB in our sample led to take data from the literature regarding potential complications of bacteremia. Therefore, CEA represents a partially literature-derived model. Fourth, the numbers of discounted life years gained were likely to overestimate real gains in life years because a large share of patients with suspected bacteremia have a clinical condition different from staphylococcal CRBSI that can potentially lead to death before reaching the general population’s expected age of death. Similarly, no attempt was done to estimate gains in quality-adjusted life years. The heterogeneity of patients’ original health condition would have made such calculation pointless. However, given that the GeneXpert test was found to be highly cost-effective in terms of unadjusted gains of life years, adjustments for quality are unlikely to change the qualitative results. Finally, the life expectancy figures could be overestimated, because large share of patients with CRBSI have a clinical condition (different from staphylococcal CRBSI) that can potentially lead to death before reaching the general population’s expected age of death. However, given that the bias applies to the estimated life expectancy obtained with both tests, the change in life years of using GeneXpert compared with BC is not likely to suffer a significant bias.

In conclusion, GeneXpert can be used directly with whole blood sample obtained through an infected catheter for the detection of *S*. *aureus* and MR-CoNS in approximately 1 h after sampling. In addition, GeneXpert is cost-effective, especially in settings with a high prevalence of staphylococcal CRBSI. Finally, this test has a potential to identify cases not detected by BC, probably due to ongoing antibiotic treatment. However, further studies are necessary to confirm this finding.

## Supporting Information

S1 File Economic evaluation of the use of GeneXpert to detect staphylococcal catheter-related bloodstream infection (CRBSI). (DOCX)
