# Supplementary material for: Rapid Diagnosis of Staphylococcal Catheter-Related Bacteraemia in Direct Blood Samples by Real-Time PCR
Source: PLoS One. 2016 Aug 29;11(8):e0161684. doi: 10.1371/journal.pone.0161684 (PMC5003366; doi:10.1371/journal.pone.0161684)
Supplement: S1 File — (DOCX) [file pone.0161684.s001.docx]

**Supplemental Material**

**Economic evaluation of the use of GeneXpert to detect staphylococcal catheter-related bloodstream infection (CRBSI).**

Base case parameter values, sources and assumptions for sensitivity analysis are described in **Table S1**. Base case values correspond to actual values observed in the present study or in the mentioned literature. Expected life years were defined as the difference between life expectancy of the Spanish population and the average age of patients with suspected CRBSI. For the sensitivity analysis, most of the variables were assumed to follow a binomial distribution (defined by the appropriate number of observations and events). The high and low values used in the deterministic sensitivity analysis correspond to the 95% confidence interval boundaries. In the case of the prevalence rate, low and high values (10% and 50%, respectively) were chosen in order to estimate the effects on health (and costs) that would occur if GeneXpert were implemented in hospitals with those specific prevalence rates. In the probabilistic sensitivity analysis, the prevalence was assumed to have a discrete uniform distribution for values {0.1, 0.2, 0.3, 0.4, 0.5}.

**Use of resources and costs data**

The use of resources corresponds to the standard treatment of patients with suspected CRBSI. Resources considered for the costs analysis include BC costs, GeneXpert kit, technical staff, catheters, wide-spectrum and specific antibiotics, imaging (abdominal ultrasound, transthoracic echocardiogram and PET) in case of haematogenous complication and the use of intensive care unit (ICU) also in case of complication.

Doses of antibiotics, type and quantity of images and the number of days spent in ICU were obtained from hospital’s clinicians (**Table S1**). Costs of resources were obtained from hospital’s administrative staff (**Table S2**).

In the probabilistic sensitivity analysis prices were assumed to have a lognormal distribution with expected price equal to base case values and scale parameter equal to 0.5. These assumptions imply that roughly a 90% of simulated prices are between 40% and 200% of base case prices.

**Sensitivity analysis**

In addition to the deterministic analysis described in the main text, a probabilistic sensitivity analysis through Monte Carlo simulations was performed. A simulation consisted in a simultaneous random draw of each parameter assuming the distributions described in **Tables S1** and **Table** **S2**. In each simulation effects on outcomes and cost of treatment per patient were computed for different prevalence levels. A total number of 10,000 simulations were performed.

The simulations showed that the mean incremental effect of GeneXpert on expected life years ranged from 0.11 when the joint prevalence of *S. aureus* and MR-CoNS was 10% to 0.55 years when the joint prevalence was 50% (**Fig S1**). The mean *incremental cost* of using GeneXpert varied from 41.2€ to -32.4€ per patient for low and high prevalence values, respectively (**Fig S2**).

At low prevalence (10%), the cost-effectiveness incremental ratio (ICER) was lower than 1,000€ per life year gained in 81.6% of the simulations and it dominated BC (i.e. it had lower cost per patient and better health outcomes) in 2.5% of the simulations. In the remaining cases, the ICER was higher than 1,000 € per patient (14.1% of the simulations) or GeneXpert was dominated (1.8%) by BC (**Fig S3**). When prevalence was high (50%) the ICER was lower than 1,000€ per life year gained in 32.1% of the simulations and GeneXpert dominated BC in a further 65% of the simulations (**Fig S4**).

**Table S1. Parameters values, sources and assumptions.**

| **Variable** | **Base case** | **Source** | **Distribution for Monte Carlo Simulations** | **Deterministic sensitivity analysis** | |
| --- | --- | --- | --- | --- | --- |
|  |  |  |  | **Low value** | **High value** |
| Sensitivity | 0.875 | Present study | Binomial | 0.62 | 0.98 |
| Specificity | 0.921 | Present study | Binomial | 0.84 | 0.97 |
| Prevalence | 0.174 | Present study | Discrete Uniform | 0.10 | 0.50 |
| Probability of complication if catheter is not removed at t = 0^a^ | 0.26 | Assumption based on literature[[1](#_ENREF_1)] | Binomial | 0.13 | 0.42 |
| Probability of complication if catheter is removed at t = 0^a^ | 0.11 | Assumption based on literature[[1](#_ENREF_1)] | Binomial | 0.08 | 0.15 |
| Probability of death (conditional on complication) | 0.70 | Assumption based on literature[[1](#_ENREF_1), [2](#_ENREF_2)] | Binomial | 0.56 | 0.86 |
| Expected life years of Spanish population at patient’s average age | 25.1 | National Statistics Institute and the present study | Normal | 21.9 | 28.3 |
| Wide-spectrum antibiotic (days) | 0 or 2^b^ | Hospital’s clinicians | Not varied in the sensitivity analysis | | |
| Narrow-spectrum antibiotic (days) | 0 or 15^c^ |  |  |  |  |
| BC (vials) | 4 |  |  |  |  |
| GeneXpert | 1 |  |  |  |  |
| Intensive care unit (days) | 1 |  |  |  |  |
| PET | 1 |  |  |  |  |
| Abdominal ultrasound | 1 |  |  |  |  |
| Transthoracic echocardiogram | 1 |  |  |  |  |

^a^at t = 0 : moment at which a patient with suspected CRBSI is detected.

^b^0 days if GeneXpert is performed and it gives a positive result and 2 days in any other case.

^c^Depending on tests results.

**Table S2. Cost data.**

| **Item** | **Price (€)** |
| --- | --- |
| Broad-spectrum antibiotic (per day) | **69** |
| Narrow-spectrum antibiotic (per day) | **49** |
| Central venous catheter (average) | **22.8** |
|  | |
| **BC (per vial):** | **14.94** |
| *Vial* | 0.94 |
| *Technical staff (20 min per vial)* | 7.00 |
| *Microbiologist (10 min per vial)* | 7.00 |
|  | |
| **GeneXpert (per sample):** | **67.20** |
| *Reagents* | 54.95 |
| *Technical staff (15 min per sample)* | 5.25 |
| *Microbiologist (10 min per sample)* | 7.00 |
| **Additional costs if complications:** | |
| One day of intensive care unit | **555** |
| One PET | **700** |
| One abdominal ultrasound | **110** |
| One transthoracic echocardiogram | **355** |


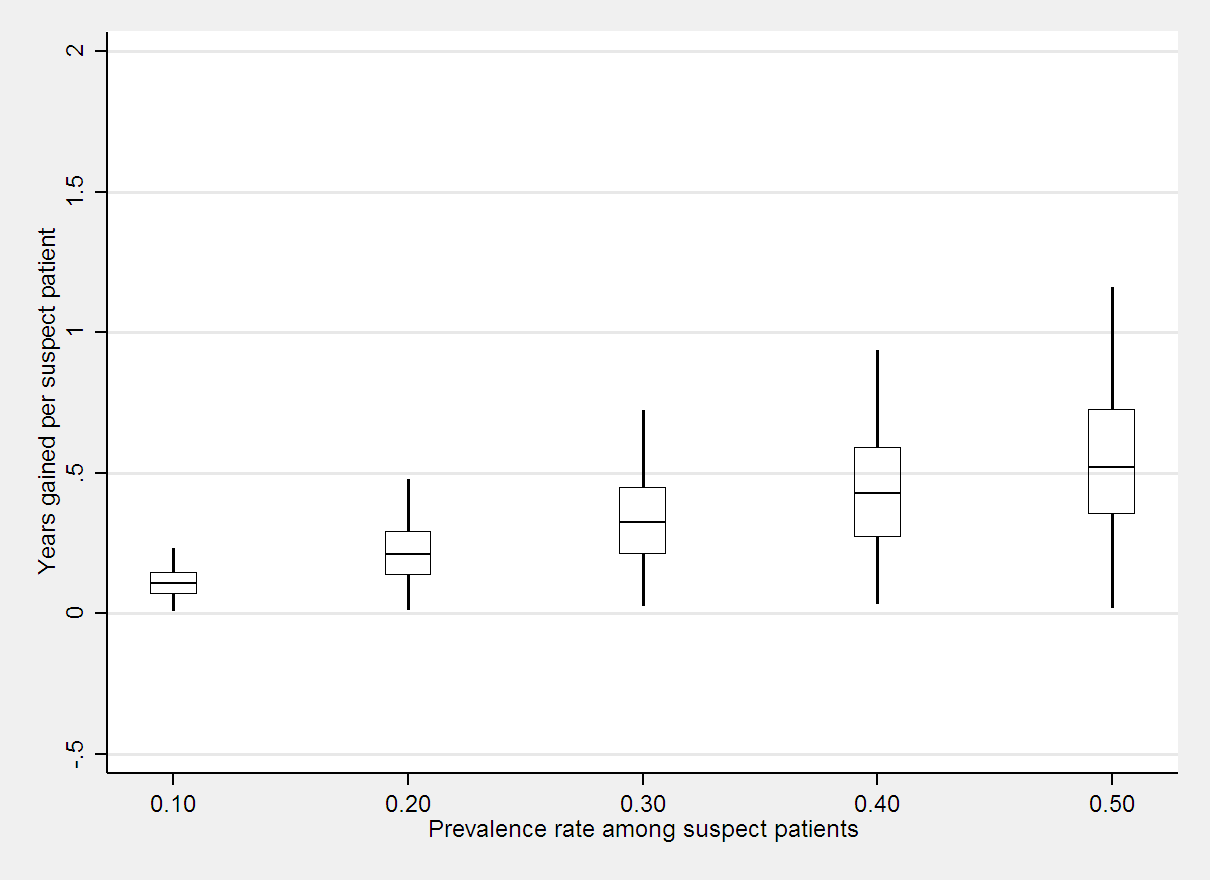


**Fig S1.** **Life years gained, by prevalence of staphylococcal CRBSI.**


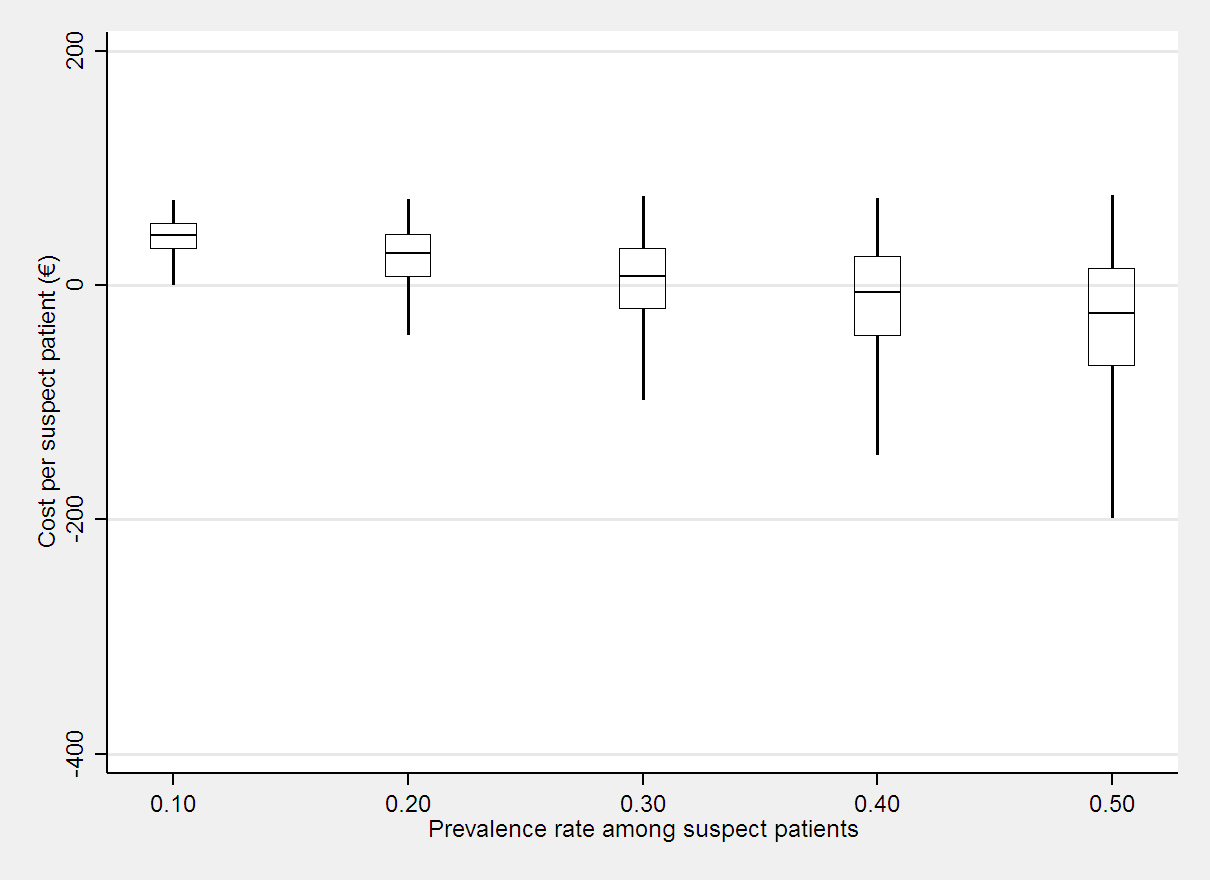


**Fig S2.** **Incremental cost, by prevalence of staphylococcal CRBSI.**


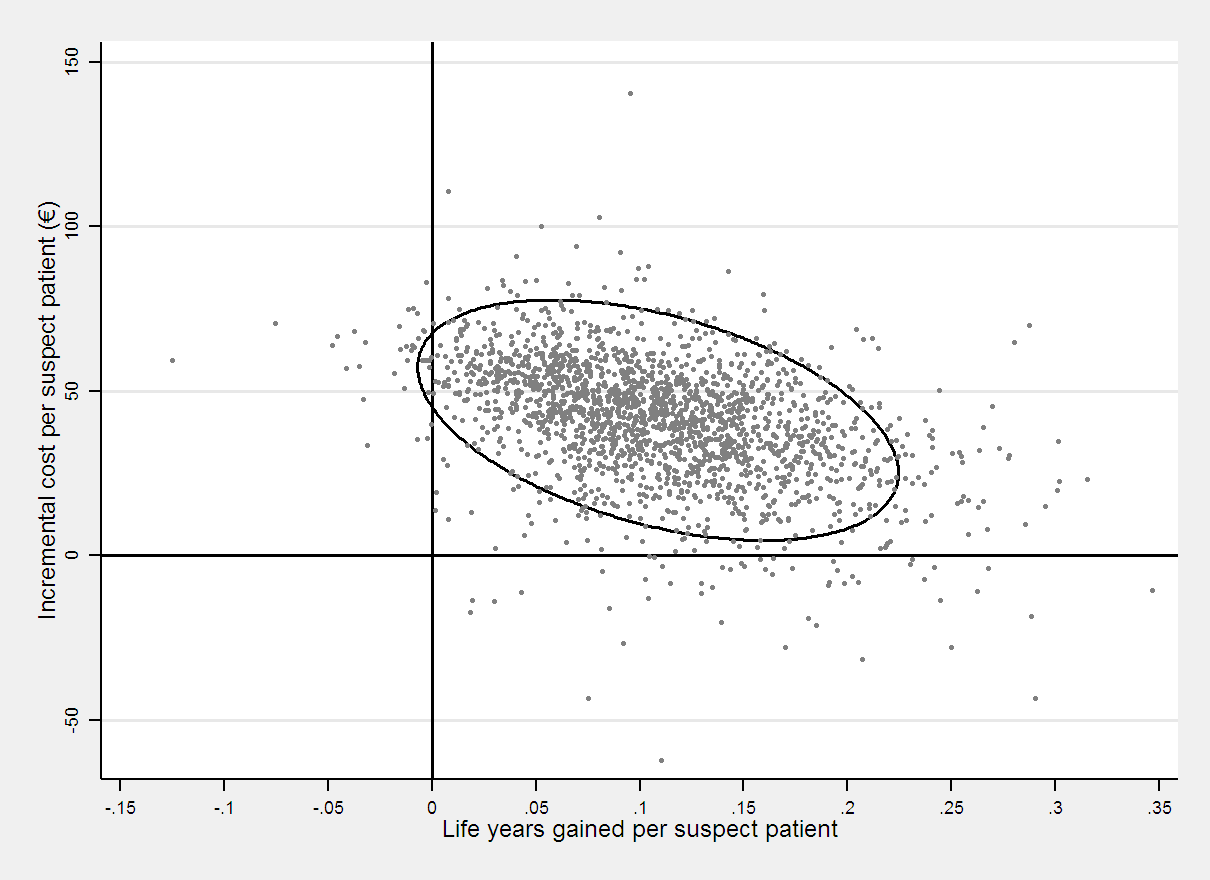


**Fig S3. Cost-effectiveness simulations at the staphylococcal CRBSI prevalence of 10% (95% confidence surface).**


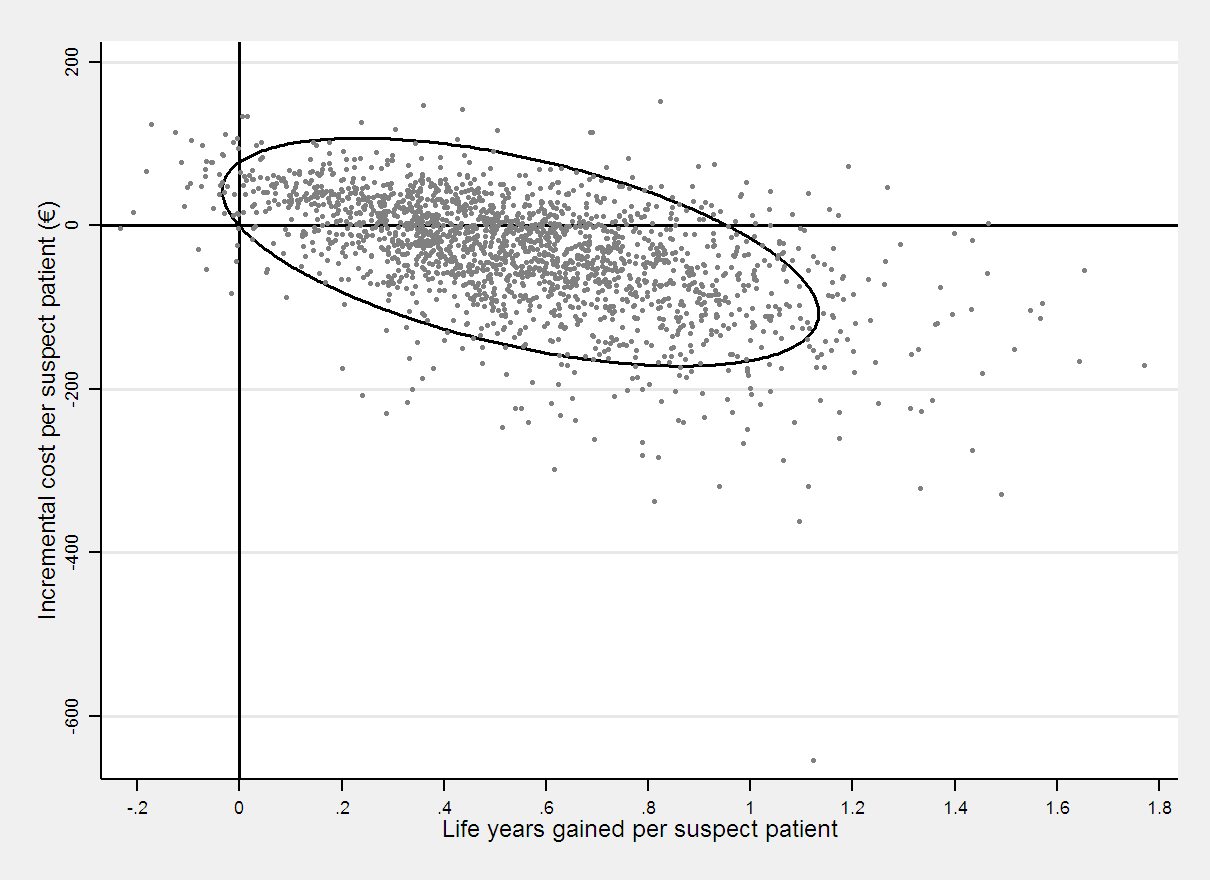


**Fig S4.** **Cost-effectiveness simulations at the staphylococcal CRBSI prevalence of 50% (95% confidence surface).**

**REFERENCES**

1. Fowler VG, Jr., Justice A, Moore C, Benjamin DK, Jr., Woods CW, Campbell S, et al. Risk factors for hematogenous complications of intravascular catheter-associated *Staphylococcus aureus* bacteremia. Clin Infect Dis. 2005; 40: 695-703. doi: 10.1086/427806 PMID: 15714415

2. Cosgrove SE, Qi Y, Kaye KS, Harbarth S, Karchmer AW, Carmeli Y. The impact of methicillin resistance in *Staphylococcus aureus* bacteremia on patient outcomes: mortality, length of stay, and hospital charges. Infect Control Hosp Epidemiol. 2005; 26: 166-174. doi: 10.1086/502522 PMID: 15756888
